# Possible Association of Telomere Length With Sleep Duration. A Preliminary Pilot Study in a Sicilian Cohort with Centenarians

**DOI:** 10.37825/2239-9754.1031

**Published:** 2021-12-23

**Authors:** Anna Aiello, Giulia Accardi, Sawan Alì, Calogero Caruso, Maxine Chen, Immaculata De Vivo, Mattia Emanuela Ligotti, Giovanni Scapagnini, Sergio Davinelli, Giuseppina Candore

**Affiliations:** a Laboratory of Immunopathology and Immunosenescence, Department of Biomedicine, Neuroscience and Advanced Diagnostics, University of Palermo, Palermo, Italy; b Department of Medicine and Health Sciences “V. Tiberio”, University of Molise, Campobasso, Italy; c Department of Epidemiology, Harvard T.H. Chan School of Public Health, Boston, MA, USA

**Keywords:** Ageing, Centenarian, Relative telomere length, Sleep duration

## Abstract

Telomere length (TL) is considered a biomarker of ageing although this topic is still debated. Also, sleep pattern changes are physiological part of the normal ageing process. In fact, it is widely recognized that sleep duration declines with age, leading to dysregulation of circadian rhythms. The aim of our study was to analyse the possible association of sleep duration with TL in a sample of 135 subjects with ages ranging from 20 to 111 years, recruited from Palermo and neighbouring municipalities in Sicily (Italy). Preliminary data suggest that relative TL (RTL) decreases with age in both men and women. However, at older ages, the difference between men and women tends to narrow. Nonagenarian and centenarian women do not show RTL values significantly different from those observed in adult and old women (40–89 years aged). Moreover, to analyse the relationship between TL and sleep, we stratified sleep duration into greater or lesser than 8-h periods. We found that centenarians, who daily sleep 8 hours or more, have longer RTL than centenarians who sleep fewer than 8 hours. Although the relatively small sample size of centenarians, we provide preliminary evidence that sleep duration may affect the RTL of centenarians. To the best of our knowledge, this is the first study to examine the relationship between centenarians, RTL and sleep duration. Further studies with greater sample size of centenarians are required to replicate and extend these data.

## 1. Introduction

Telomeres are specialized dynamic nucleoprotein structures that contribute to maintain the structural integrity of chromosomes [1]. Telomeres are actively maintained in human germ line cells and embryonic stem cells by action of telomerase. Telomeres shorten when somatic cells divide due to insufficient telomerase expression [2]. Therefore, the length of telomeres decreases with age due to incomplete replication of DNA ends with each cell division. On average, human telomeres lose 50 to 100 base pairs per mitotic division [3]. This telomere shortening results from a combination of a failure to completely replicate the ends of linear DNA molecules, *i.e*., the “end replication problem,” and the processing of DNA that occurs on the ends of linear chromosomes [2]. Telomere length (TL) is a reliable indicator of intrinsic biological age and a surrogate for the mitotic clock. The role of telomeres in human biology has been studied since the early 1990s because telomere attrition has been implicated both in ageing and in age-related diseases [4]. Telomere length is a key determinant of the normal lifespan of a cell and is closely connected with the cell cycle and replicative age [5]. Telomeres that are too short lead to a decrease in functional cells and contribute to overall tissue and organ dysfunction [6]. Short telomeres are commonly found in several age-related conditions as a biomarker of disease onset and related outcomes [7]. Indeed, the rate of telomere loss is affected by multiple environmental factors other than the mitotic replication rate. There is evidence that lifestyle-related factors, such as tobacco abstinence, moderate physical activity, mental well-being, and healthy dietary patterns, may reduce telomere attrition throughout human life likely because there is a better control of oxidative stress, so reducing DNA damage [8–10]. Indeed, at a molecular level, due to the high guanine-cytosine content and long stretches of repetitive DNA, telomere sequences are particularly vulnerable to oxidative and inflammatory conditions. In particular, the formation of 8-oxodG at the GGG triplet in telomere sequence induced by oxidative stress could participate in acceleration of telomere shortening [11]. Moreover, in older people, low-grade systemic inflammation *(i.e.*, inflamm-ageing) is strictly connected to oxidative stress and both are linked to the telomere shortening and onset of age-associated diseases [12].

Sleep is a reversible and recurrent state of reduced responsiveness to external stimulation that is accompanied by important restorative functions, such as metabolic recovery, memory consolidation, or synaptic scaling [13–15]. Many sleep-related issues occur with increasing frequency among older people. However, age-related sleep changes are probably related to a decreased ability to sleep, rather than a decreased need for sleep [16]. With ageing, various factors deteriorate the normal sleep process, including cortical thinning, white matter degeneration, neurotransmitter dysregulation, and circadian disorganization [17]. Despite the great scientific interest to identify mechanistic pathways between sleep and ageing, only a little attention has been paid to the role of TL. However, there is some evidence on the relationship between short leukocyte telomeres and short sleep duration, poor sleep quality, and insomnia [18–20]. It is feasible that sleep disturbances are associated with several age-related alterations in biological processes that can interfere with TL maintenance, since normal sleep patterns have been showed to play a crucial role in the homeostatic regulation of inflammatory and oxidative pathways known to negatively influence TL [9,21].

To better understand the possible association between sleep duration and TL maintenance, we investigated sleep habits and TL in a population of 135 subjects with ages ranging from 20 to 111 years. For the definition of sleep habits, we choose an 8-h cut-off based on the midpoint of the American National Sleep Foundation’s recommendation for a 7–9 hours sleep duration for young adults and adults, and 7–8 hours of sleep for older adults [22]. For the TL assay we used DNA from blood leukocytes. Results of calculated median telomere fluorescence in granulocytes and lymphocytes of 400 normal individuals over the entire age range demonstrated that the telomere length is highly variable at any given age, showing a highly significant decline with age that is most pronounced for lymphocytes [23]. Indeed, for lymphocytes their shortened telomeres are the result of both the product of many divisions from the hemopoietic stem cells to the mature peripheral form as for granulocytes and the history of their cycles of activation, which depends on their immunobiography [24].

## 2. Materials and methods

### Participants

Participants were recruited From June 2017 to February 2019 within the project “Discovery of molecular and genetic/epigenetic signatures underlying resistance to age-related diseases and comorbidities (DESIGN)”, funded by the Italian Ministry of Education, University and Research. The Ethics Committee of Palermo University Hospital (Sicily, Italy) approved the study protocol (Nutrition and Longevity, No. 032017). The study was conducted in accordance with the Declaration of Helsinki and its amendments. All study participants (or their caregivers) gave their written informed consent prior to enrolment. A total of 135 healthy donors (65 men and 70 women) aged between 20 and 111 years, were recruited in Palermo and neighbouring municipalities in Sicily (Table 1). All study participants were Sicilians, selected on the basis of their health status. We selected healthy people, considering the age physiological deterioration of organs and systems, including deafness, visual problems, and if they have no more than one invalidating condition. Moreover, we included cognitive-performant individuals only (although not completely). Thus, we excluded people with chronic invalidating diseases, such as neoplastic and auto-immune ones, as well as with acute disease, such as infectious, and with severe dementia. Before enrolment, the participants or their caregivers signed an informed consent form to release sensitive data. To respect the privacy, all other donors were identified with an alphanumeric code. A well-trained team of experts from the University of Palermo administered to participants a detailed questionnaire to collect anamnestic data including sleep habits. For oldest people, sleep habits were described by children or caregivers. A database was created to handle the collected information. For more information about recruitment criteria, please see ref. n. [25]. The participants underwent venipuncture in the morning, after a fasting period of 12 hours. The blood was collected in specific tubes containing ethylene diamine tetraacetic acid (EDTA) or no additives. Serum was separated by blood centrifugation of dry tubes and stored at −80 °C before use. Data were analysed using R software version 3.5.3.

### Determination of telomere length

Genomic DNA was extracted from leukocytes with a commercial kit (QIAmp DNA Mini Kit, Qiagen). Relative telomere length (RTL) was determined at the Dana Farber/Harvard Cancer Center Genotyping & Genetics for Population Sciences Facility (Boston, MA, USA), using a modified, high-throughput version of the real-time quantitative PCR-based telomere assay that was run on the Applied Biosystems 7900HT Sequence Detection System (Foster City, CA) [26–28]. Fifteen ng of genomic DNA was required for the protocol. The average RTL was determined as the copy-number ratio between telomere repeats and a single-copy (36B4) reference gene (T/S Ratio, −ΔCt) [29,30]. Each sample was assayed in triplicate by laboratory technicians who were masked to participant characteristics; variability was assessed with inclusion of quality control (QC) samples on each plate. Leukocyte RTL is reported as the exponentiated T/S ratio corrected for a reference sample. Although this assay measures RTL, in work by Cawthon [26], the T/S ratio highly correlates with absolute TL provided by Southern Blot (r = 0.68–0.85; p < 0.0001) [26]. QC samples (10%) were included in each batch and each QC sample was evenly split into halves. Based on the repeated measurements of these QC samples, the coefficient of variation (CV) for evaluating the performance of TL assays was calculated. The CV range was 0.2%–3.1% for the single-copy gene assay, 0.6%–2.1% for the telomere assay, and 10.8–16.0% for the exponentiated T:S ratio. Because TL was assayed in several batches, we log-transformed the RTL to minimize the impact of potential batch shifts on its measurements across different batches, and then calculated a Z-score of RTL by standardizing the RTL compared to the mean within each individual study.

## 3. Results

**Fig. 1:**
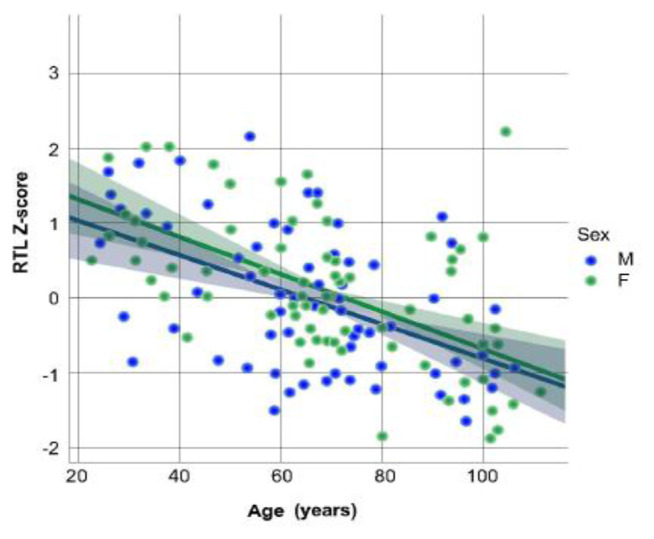
Relative telomere length (RTL) Z-score decreases with age in both men (N = 65) and women (N = 70) (p < 0.0001 by regression analysis).

Fig. 1 depicts the correlation between RTL values and age. As expected, the RTL values decrease with age in both men and women. However, the Figure shows that the regression line for women is a bit more steeply going down with older age than for men, although not significantly. It is noteworthy that young women (ages 20–39) show RTL significantly higher than those observed in the other age groups (p < 0.01 by ANOVA test), whereas nonagenarian and centenarian women do not show RTL values significantly different from those observed in adult and old women (ages 40–89).

**Fig. 2:**
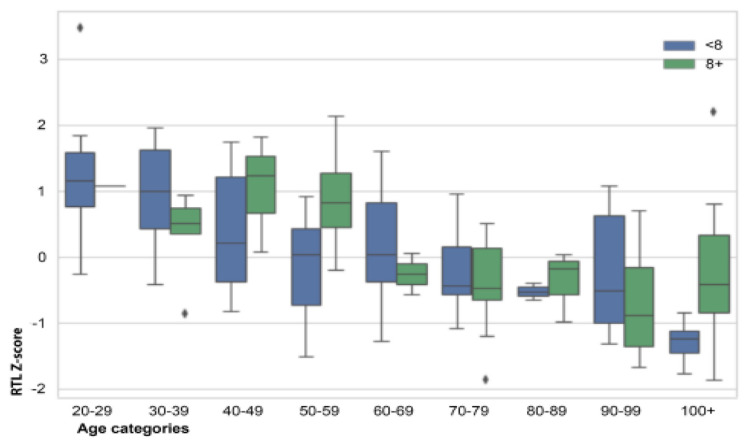
Relative telomere length (RTL) Z-score of the nine age groups of men and women divided according to sleep duration. Small black diamonds refer to outliers (for RTL-Z score).

To analyse the relationship between TL and sleep, we divided the subjects into nine age category groups and categorized sleep duration into <8 hours and ≥8 hours (Table 1). Fig. 2 shows that centenarians who sleep 8 or more hours per day have longer RTL than centenarians who sleep fewer than 8 hours per day. However, this interesting datum was statistically not significant, probably because of the small sample size of centenarians. Otherwise, no obvious trend based on sleep duration was observed. Plotting hours of sleep of each participant against the TL Z-scores no significance was obtained. Furthermore, narrative report on sleep duration might bias estimates of sleep duration.

## 4. Discussion

Telomeres are repetitive sequences of DNA at the ends of chromosomes that suffer attrition with each mitotic division of a somatic cell. Moreover, short telomeres represent a marker of the cumulative load of inflammation and oxidative stress. Therefore, telomeres can be considered as a memento of previous cell divisions and DNA damage. Indeed, although ageing is a multifactorial and complex event, healthy ageing and longevity are believed to be associated with longer telomeres and lower inflammation profiles among older individuals. However, despite accumulating data on a strong interconnection between telomerase regulation/activity and inflammation, the mechanistic details and molecular pathways of this connection have not yet been discovered [31].

Older adults with high levels of inflammatory activity may be at increased risk for accelerated leukocyte telomere shortening, and those with short TL may have increased risk for diseases with an inflammatory aetiology [3,8–10,32]. Nevertheless, clear causative proof of telomeres in age-related diseases is still lacking. Indeed, it should be necessary to determine whether telomere shortening is a cause or an effect of ageing and related diseases [33]. However, telomere-driven cell senescence can limit the regenerative capacity of tissues by compromising the function of tissue-specific stem and progenitor cells and accelerating ageing. Furthermore, the accumulation of senescent cells may be responsible for organ failure due to the depletion of the organ renewal capacity associated with ageing [23,33]. Accordingly, it has been demonstrated that individuals who lead a healthy lifestyle have been shown to have longer telomeres than those who do not adhere to this lifestyle [8,9]. Conversely, a large study has demonstrated that short telomeres are associated with a higher risk of all-cause mortality [18].

Concerning oxidative stress, ageing is associated with an increase in pro-oxidant factors and a decrease in antioxidant mechanisms. Oxidative stress plays an important role in determining and maintaining the typical low-grade inflammation, which in turn contributes to oxidative stress. However, in several groups of centenarians, some indexes of oxidative stress have been demonstrated to be lower than in older subjects [34].

Our recent surveys performed in Sicilian centenarians, have demonstrated a close adherence the anti-inflammatory Mediterranean diet only in young age. However, their diet was always an anti-inflammatory one. The range values of oxidative markers in Sicilian centenarians have been shown to be included in the value range of young people. Moreover, the total antioxidant capacity is lower in young people than in the other groups and that the highest values are observed in nonagenarians [24,35–37]. So it is not surprising that in our samples, centenarian women have RTL not significantly different from that of older female adults. Moreover, the RTLs of two semisuper and super-centenarian sisters have been shown to fit in the average plus/minus standard deviation of 60–69 Sicilian women [36].

Sleep plays a role in the regulation of synaptic plasticity and protein synthesis. Its deprivation may alter protein synthesis and synaptic plasticity through oxi-inflammatory mechanisms [38]. In this regard, some evidence suggests that telomere length may be related to sleep duration [18–20]. Moreover, also the environment may be responsible for both shorter telomere length and reduced sleep duration: i.e., stress, a known agent of telomere shortening [9].

However, a population-based study of young and midlife people shows no correlation between sleep duration and RTL, contrary to previous findings which showed an association between sleep duration and shorter telomeres in children and in adults as well as in women under the age of 50, but not among those over 50 [20,39–42]. The authors suggest that, in healthy adults, sleep characteristics play only a limited role in the shortening of telomeres. Thus, sleep–telomere associations should be a late-life occurrence or occur only in the presence of other morbidities [39]. Only one report supports the hypothesis that sleep is a relevant factor affecting telomere length in older adults [43].

To the best of our knowledge, this study is the first report to study a relationship between RTL and sleep duration in a group of centenarian subjects. In our cohort, centenarians who slept 8+ hours per day had longer RTL than subjects from the same age group who slept less than 8 hours per day. However, this finding was not statistically significant due to the small sample size of centenarians and nonage-narians. To achieve adequate statistical power, future cohort studies should include a larger sample of long-living individuals.

## Figures and Tables

**Table 1:** Participants divided by age group and sleep (hours) duration.

Sleep	20–29	30–39	40–49	50–59	60–69	70–79	80–89	90–99	100+	Total
<8	9	9	5	10	27	16	2	9	8	95
>8	1	4	3	4	2	9	3	7	7	40
Total	10	13	8	14	29	25	5	16	15	135
